# Type 2 diabetes in the Democratic Republic of Congo: an urgent need for a management framework

**DOI:** 10.1093/heapro/daad139

**Published:** 2023-12-01

**Authors:** Jean-Pierre Fina Lubaki, Olufemi Babatunde Omole, Joel Msafiri Francis

**Affiliations:** Department of Family Medicine and Primary Care, School of Clinical Medicine, Faculty of Health Sciences, University of the Witwatersrand, Johannesburg, South Africa; Department of Family Medicine and Primary Care, Protestant University of Congo, Kinshasa, Democratic Republic of the Congo; Department of Family Medicine and Primary Care, School of Clinical Medicine, Faculty of Health Sciences, University of the Witwatersrand, Johannesburg, South Africa; Department of Family Medicine and Primary Care, School of Clinical Medicine, Faculty of Health Sciences, University of the Witwatersrand, Johannesburg, South Africa

**Keywords:** policy, strategies, interventions, glycaemic control, type 2 diabetes

## Abstract

Glycaemic control is of one the main goals for managing type 2 diabetes. In sub-Saharan Africa and the Democratic Republic of the Congo, studies have reported alarming poor control rates. Patients with poor glycaemic control are exposed to complications leading to high cost of care and deteriorated quality of life. In recent studies by our group, we have demonstrated that poor glycaemic control is high and driven by proximal (individual) and distal (structural) factors in Kinshasa, Democratic Republic of the Congo. Financial constraints impacted many aspects of care at multiple levels from the Government to persons living with diabetes. Financial constraints prevented good preparation, organization and access to diabetes care. Difficulties in implementing lifestyle changes, lack of health literacy and limited healthcare support were also contributing to poor glycaemic control. Through a Delphi study, a group of experts reached a consensus on five potential strategies for improving glycaemic control in the Democratic Republic of Congo as follows: changing the healthcare system for better diabetes care extended to other noncommunicable diseases, ensuring consistent financing of the healthcare, augmenting the awareness of diabetes among the general population and the persons living with diabetes, easing the adoption of lifestyle modifications and reducing the burden of undiagnosed diabetes. This paper reflects on the urgent need for an improved management framework for diabetes care in the Democratic Republic of the Congo. Specifically, the Government needs to increase the investment in the prevention and treatment of noncommunicable diseases including diabetes.

**Contribution to Health Promotion**

- Description of factors driving poor glycaemic control for accurate drafting of interventions in Kinshasa, Democratic Republic of Congo.
- Identification of strategies of interventions for improving glycaemic control in Kinshasa, Democratic Republic of Congo.
- Description of implications of the research findings for sub-Sahara Africa.

## BACKGROUND

The burden of diabetes mellitus is on the increase and this had made it a significant public health problem globally (IDF, 2022a). In 2021, it was estimated that 1 in 22 adults in sub-Saharan Africa were living with diabetes, which was responsible for 416 000 deaths (IDF, 2022a). This figure is only a part of the diabetes burden as in Africa, a considerable proportion of people with diabetes are undiagnosed (Ogurtsova *et al.*, 2022). Moreover, the WHO African Region is expected to experience the greatest increase in diabetes of all regions by 2045 by 134% (IDF, 2022a). Recent data estimated that 5.8% of adult population lives with diabetes in the Democratic Republic of Congo (DRC) (IDF, 2022b). This increase in the prevalence of type 2 diabetes is associated with modifiable risk factors, such as urbanization and obesity (Motala *et al.*, 2022).

Despite its increasing importance, diabetes is not receiving the deserved attention in many countries in sub-Saharan Africa, including the DRC. Diabetes care experiences several challenges including lack of organization in the health system, insufficient financing for diabetes care and limited information on the diabetes complications and the genetics (Atun *et al.*, 2017; Pastakia *et al.*, 2017; Motala *et al.*, 2022).

Due to inadequate diabetes care, studies have shown that less than one third of the persons with diabetes reached glycaemic targets in sub-Saharan Africa and in the cosmopolitan city of Kinshasa, DRC (Lubaki *et al.*, 2022; Lubaki *et al.*, 2023a). Glycaemic control is particularly important in the management of diabetes because it delays or prevents the onset of diabetes-related complications (Bin Rakhis *et al.*, 2022). Diabetes complications negatively impact the quality of life and lead to premature mortality. In May 2022, WHO Members States recommended the strengthening and monitoring of diabetes response as part of the noncommunicable diseases programmes, the 75th World Health Assembly set five targets by 2030 including 80% of those living with diabetes should have good control of glycaemia (WHA, 2022). The other targets were: 80% of people living with diabetes are diagnosed, 80% of people with diagnosed diabetes have good control of blood pressure, 60% of people with diabetes of 40 years or older receive statins and 100% of people with type 1 diabetes have access to affordable insulin and blood glucose self-monitoring.

Due to a lack of practical framework for improving glycaemia control among persons living with diabetes in the DRC, we conducted four sub-studies to explore ways of improving glycaemic control in the DRC. The first sub-study was a systematic review and meta-analysis on the prevalence and factors driving glycaemic control in sub-Saharan Africa (Lubaki *et al.*, 2022); this review showed high prevalence of poor glycaemic control among persons with type 2 diabetes in sub-Saharan Africa and highlighted six categories of factors driving glycaemic control in one or another direction: sociodemographic, lifestyle, clinical, adherence to treatment plans, treatment modalities and glycaemic control optimization interventions. The second sub-study was a mixed-methods cross-sectional study in Kinshasa, DRC which provided quantitative and qualitative perspectives of the factors associated with poor glycaemic control (Lubaki *et al.*, 2023a, b). The third sub-study described the perspectives of healthcare providers and patients with type 2 diabetes on ways to improve glycaemic control in Kinshasa (Lubaki *et al.*, 2023c), and finally, an electronic anonymous two-round Delphi study built a consensus on interventions for improving glycaemic control in Kinshasa, DRC (Lubaki *et al.*, 2023d). Based on the findings of the previous studies of our project, this paper gives a summary of the determinants identified for poor glycaemic control and highlights policy recommendations for improving glycaemic control among patients with type 2 diabetes in Kinshasa, DRC.

## DETERMINANTS OF POOR GLYCAEMIC CONTROL

In the DRC, the five main determinants for poor glycaemic control among persons with type 2 diabetes were: financial constraints, lack of affordable healthcare coverage, difficulties in implementing lifestyle changes, lack of health literacy and limited healthcare and social support.

### Financial constraints

Diabetes mellitus is a lifelong and costly disease requiring many life adjustments including financial ones to enable reliable and consistent funding of care (Mouchereau *et al.*, 2019; Mutyambizi *et al.*, 2019). The economic situation in the DRC is very precarious (World Bank, 2023). In 2019, the country spent only 4% of its Gross Domestic Product on healthcare services (WHO, 2023). Unemployment is very high and the population’s purchasing power very low (World Bank, 2023). Our study participants reported not having sufficient financial means to cover the costs of diabetes care (Lubaki *et al.*, 2023b). Lack of financial resources impacts negatively on diabetes self-care and comprehensiveness of healthcare services for patients (Lubaki *et al.*, 2023b). Participants reported that they do not have money in time to buy medicines and could not adhere to medications as prescribed. Lack of financial resources prevented the patients to timely take their medicines, particularly insulin, for fear of hypoglycaemia when they do not have something to eat. They could also not properly monitor their glycaemia because tests have to be paid for and neither could they adhere to prescribed dietary changes needed for optimal control.

### Lack of affordable healthcare coverage

In the DRC, only a small proportion of the population is covered by health insurance (Solidar, 2016). Most persons with diabetes rely on out-of-pocket payments to cover the costs of diabetes (Laokri *et al.*, 2018). This predisposes the poor population to a vicious cycle impoverishment (Jakovljevic and Getzen, 2016) as they would not always have enough money to afford the drugs, becoming unable to take care of themselves and presenting with complications that further increase costs and disability, that subsequently hinders economic productivity and income.

### Difficulties in implementing lifestyle changes

In our qualitative exploration of the perspectives of patients (Lubaki *et al.*, 2023b), participants reported that they had difficulties in implementing lifestyle modifications. The main barrier was their work commitment that did not allow for time to engage in physical exercise at their workplaces and in most cases, make them vulnerable to eating meals that were not recommended—often fast foods. They arrived home late and had the main meal of the day very late. Patients with type 2 diabetes are often elderly, have other co-morbidities and are physically impaired, making physical exercise difficult.

### Lack of health literacy

A study in our setting showed that the knowledge of persons with type 2 diabetes was poor (Ntontolo *et al.*, 2017) due, among many reasons, to lack of awareness campaigns (Lubaki *et al.*, 2023b). The lack of information could render the population unable to seek appropriate care and in other cases to seek alternative treatment, believing that their diabetes is the result of an evil spell and that they need spiritual deliverance (Lubaki *et al.*, 2023b).

### Limited healthcare and social support

Our study participants reported that they received little support in the management of their type 2 diabetes (Lubaki *et al.*, 2023b). Their closest family members were the main source of support and this was mainly emotional support. No financial or other types of support was available. Patients living with type 2 diabetes had to rely on their own meagre and inadequate resources. There are no available aid or support from the Government for the care of patients with diabetes.

## RECOMMENDATIONS

Reflecting on the findings of the previous studies of the project, the experts in the Delphi study recommended five strategies for improving glycaemic control among patients with type 2 diabetes: changing the healthcare system for better diabetes care extended to noncommunicable diseases, ensuring consistent financing of the healthcare, augmenting the awareness of diabetes among the general population and the patients, easing the adoption of lifestyle modifications and reducing the burden of undiagnosed diabetes (Lubaki *et al.*, 2023d). For each strategy, key activities are highlighted and their rationale explained.

### Changing the healthcare system for better care of diabetes extended to other noncommunicable diseases

Achieving better glycaemic control in our environment requires changes in the way the healthcare system operates (WHO, 2010). Current strategies recommend a multidisciplinary approach to diabetes within primary health care structures in an integrated approach of managing noncommunicable diseases (WHO, 2020; WHO. Regional Office for Africa, 2022). The national program should ensure that evidence-based and context-orientated guidelines are developed and vulgarized all over the country (Lubaki *et al.*, 2023d). Healthcare staff must be trained to develop a better approach to persons with diabetes, including support for self-management (Lubaki *et al.*, 2023d). Achieving these changes must be supported by reliable data collection demonstrating the importance of diabetes and noncommunicable diseases in the country’s morbidity, mortality and economy (Pastakia *et al.*, 2017; Lubaki *et al.*, 2023d). Only the advocacy based on accurate data will allow the transformation of mind and health policy towards noncommunicable diseases, including diabetes (Atun *et al.*, 2017; Pastakia *et al.*, 2017).

### Ensuring consistent financing of the healthcare

An effort should be made by the Government to ensure consistent funding of healthcare (Pastakia *et al.*, 2017; Lubaki *et al.*, 2023d). The Government should also consider incorporating a source of healthcare funding, such as a health tax, which could support an increase in the Government’s contribution to healthcare costs. Another source of financing is the use of the taxation of tobacco, alcohol, sweetened beverages and fast food manufacturers. The search for support from the international community is an opportunity. Taking into account the growing importance, The Ministry of Health should ensure that diabetes and, by extension, the other important noncommunicable diseases in our environment are not neglected (Pastakia *et al.*, 2017). A partnership with the private pharmaceutical sector could help in this purpose. The Government could reduce taxes on medications and supplies for the care of diabetes, with a view to lowering their costs.

### Augmenting the awareness of diabetes among general population and persons living with diabetes

It is crucial to ensure that the general public is more aware of diabetes (Lubaki *et al.*, 2023d). The national program against diabetes should organize awareness campaigns for these diseases, which are becoming increasingly important. As part of this campaign, we need to take account of the need to get messages across via the popular drama on the public media platform. Community-based campaigns in public spaces, churches and schools have been used as an appropriate approach in our environment.

### Easing the adoption of lifestyle modifications

Lifestyle modifications are the cornerstone of type 2 diabetes management in Africa (Bekele *et al.*, 2020). Persons with diabetes need to adopt healthy diet, increase physical activity, reduce harmful alcohol intake and quit tobacco use (Bekele *et al.*, 2020; Power *et al.*, 2020). The critical point is to integrate lifestyle changes into the daily activities for persons living with diabetes in our setting (Lubaki *et al.*, 2023d). Advocating for the integration of the practice of physical exercise into the workplace has the potential for great impact in the DRC. Policies and regulations that control the marketing and sales of tobacco, alcohol, sweetened beverages and fast foods also need to be applied to try to control their use by the public. As far as diet is concerned, it is essential to define appropriate diets in terms of what is available locally as staple foods and patient’s contextual issues, including what is affordable for them. Given the lack of local data on how best it is to implement lifestyle in Kinshasa or in DRC, operational studies are needed to determine the best interventions for lifestyle change that takes into account the patient’s condition(s) and purchasing power.

### Reducing the burden of undiagnosed diabetes

Opportunistic screening for diabetes that targets individuals at high risk of the disease such as those with the past history of CVD, family history, obesity, concurrent hypertension and/or other cardiovascular risk factors could therefore identify new cases at early stages of the diseases when complications have not set in. Monitoring the weight gain, assessing the body mass index and measuring the waist circumference for each patient consulting in a primary healthcare facility will contribute to early screening of diabetes. Providing reliable means of laboratory diagnosis as a point of care glycosylated haemoglobin will be important to consider. With prompt lifestyle changes and treatment, early detection could ease the management at diagnosis and delay the occurrence of complications (Echouffo-Tcheugui *et al.*, 2012).

## IMPLICATIONS OF THE RESEARCH

Most countries in sub-Saharan Africa face similar challenges when it comes to caring for patients with type 2 diabetes. In a broader perspective, improving care of noncommunicable diseases including diabetes requires more involvement from the Government and the society in terms of better financing, health system preparation and prevention. A task force at the regional level could help in monitoring the progress in the diabetes care made by the countries, sharing the information on evidence-based strategies and mobilizing the resources available worldwide (Hunt *et al.*, 2021; WHO. Regional Office for Africa, 2022).
